# Supplementary figures and images for: Genome-wide analysis of fitness determinants of Staphylococcus aureus during growth in milk
Source: PLoS Pathog. 2025 Apr 9;21(4):e1013080. doi: 10.1371/journal.ppat.1013080 (PMC12011298; doi:10.1371/journal.ppat.1013080)

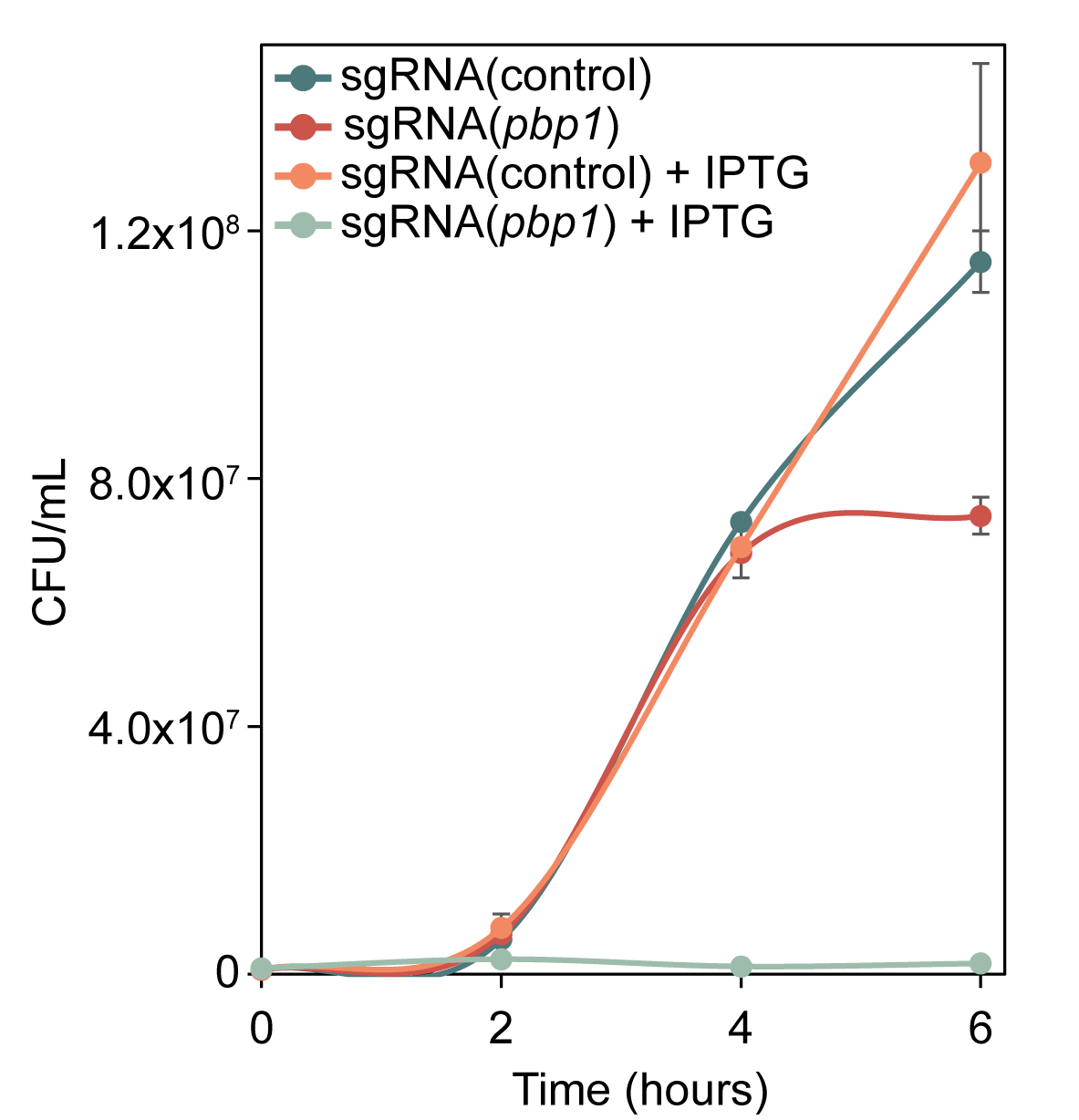

Supplement: S1 Fig — Strains were grown in UHT milk for 6 hours with or without induction with 500 µm IPTG. CFU/ml was calculated at 2-hour intervals. (TIF) [file ppat.1013080.s001.tif]

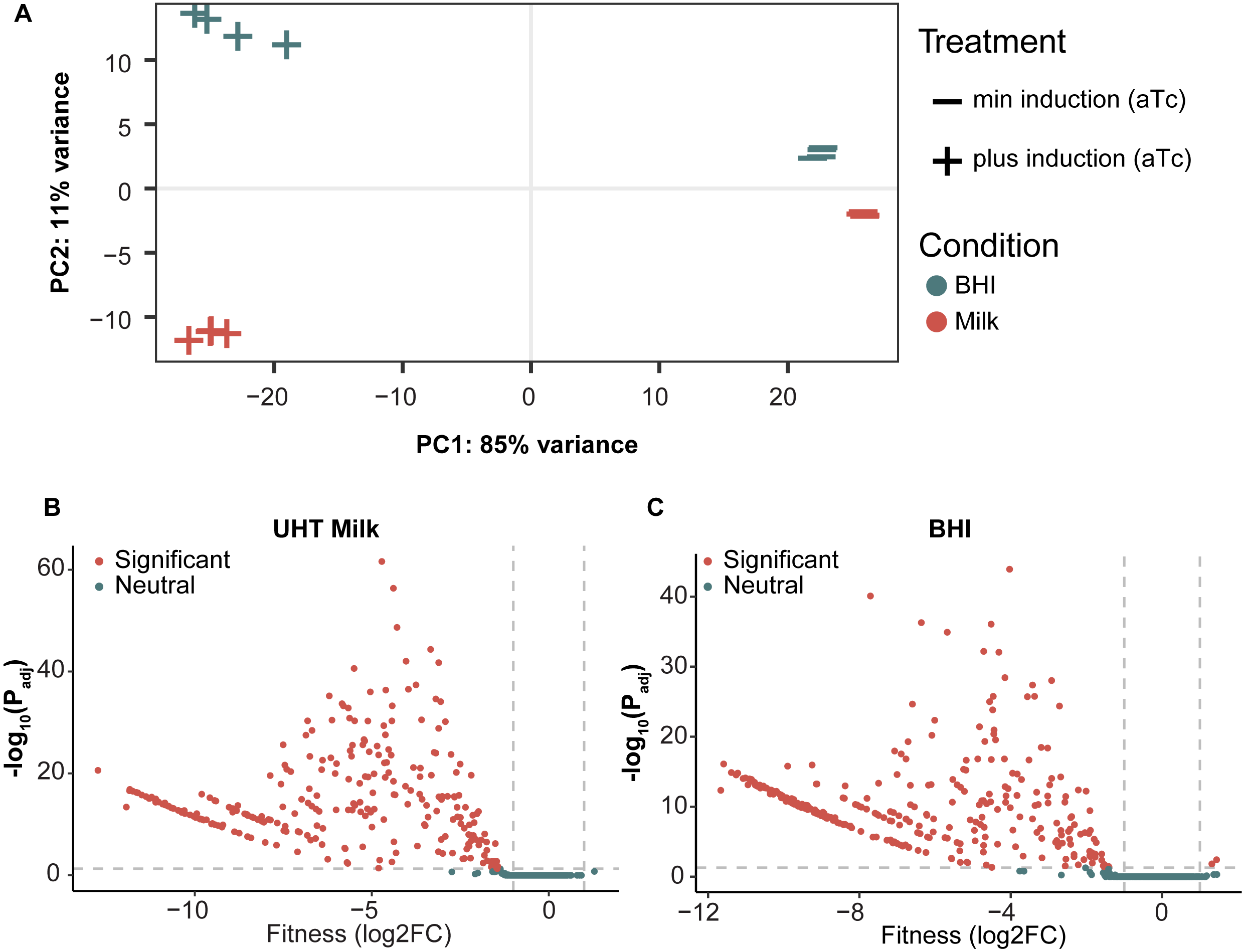

Supplement: S2 Fig — (A) Principal component analysis (PCA) of the rlog-transformed sgRNA counts. (B) Fitness effect upon dCas9 induction in UHT milk. (C) Fitness effect upon dCas9 induction in BHI. Genes with significant fitness effects (|log2FC| ≥ 1, Padj < 0.05) are depicted in red, while green points indicate neutral genes. Dashed grey lines denote the thresholds for significance (|log2FC| ≥ 1, Padj < 0.05). The data were analyzed using DESeq2 [72], with normalized counts calculated using the ‘counts()’ function, and log2FC values were shrunk using the ‘apeglm’ method [73]. (TIF) [file ppat.1013080.s002.tif]

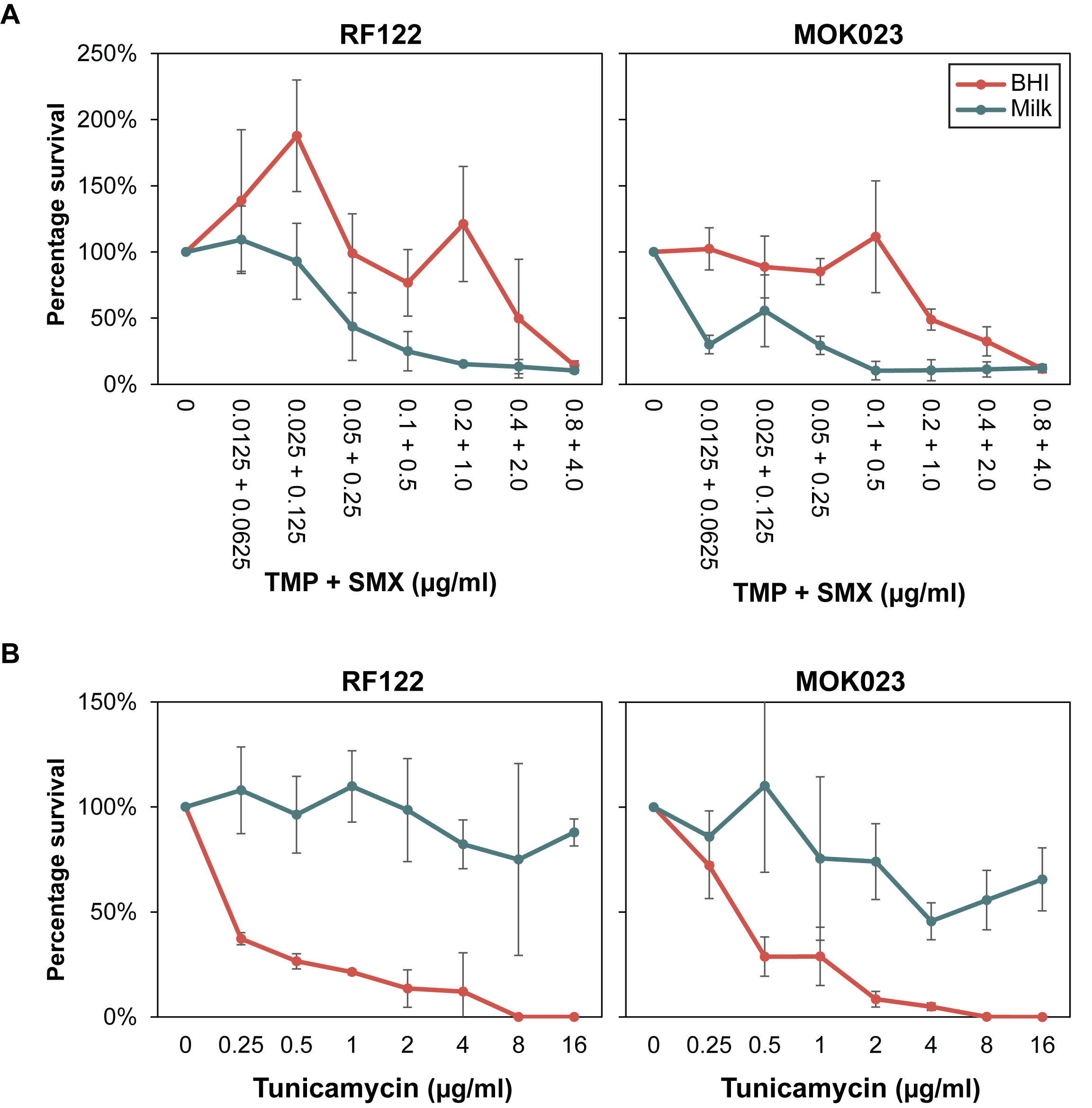

Supplement: S3 Fig — Cultures of S. aureus were diluted 1/1000 in BHI and milk containing a 2-fold dilution of antibiotics and subsequently incubated at 37 °C for 6 hours. CFU/ml was calculated from 5 µl aliquots spotted on BHI agar plates, and percentage survival was calculated compared to untreated cultures. Data represent the mean of three independent experiments, with error bars indicating standard errors. (TIF) [file ppat.1013080.s003.tif]

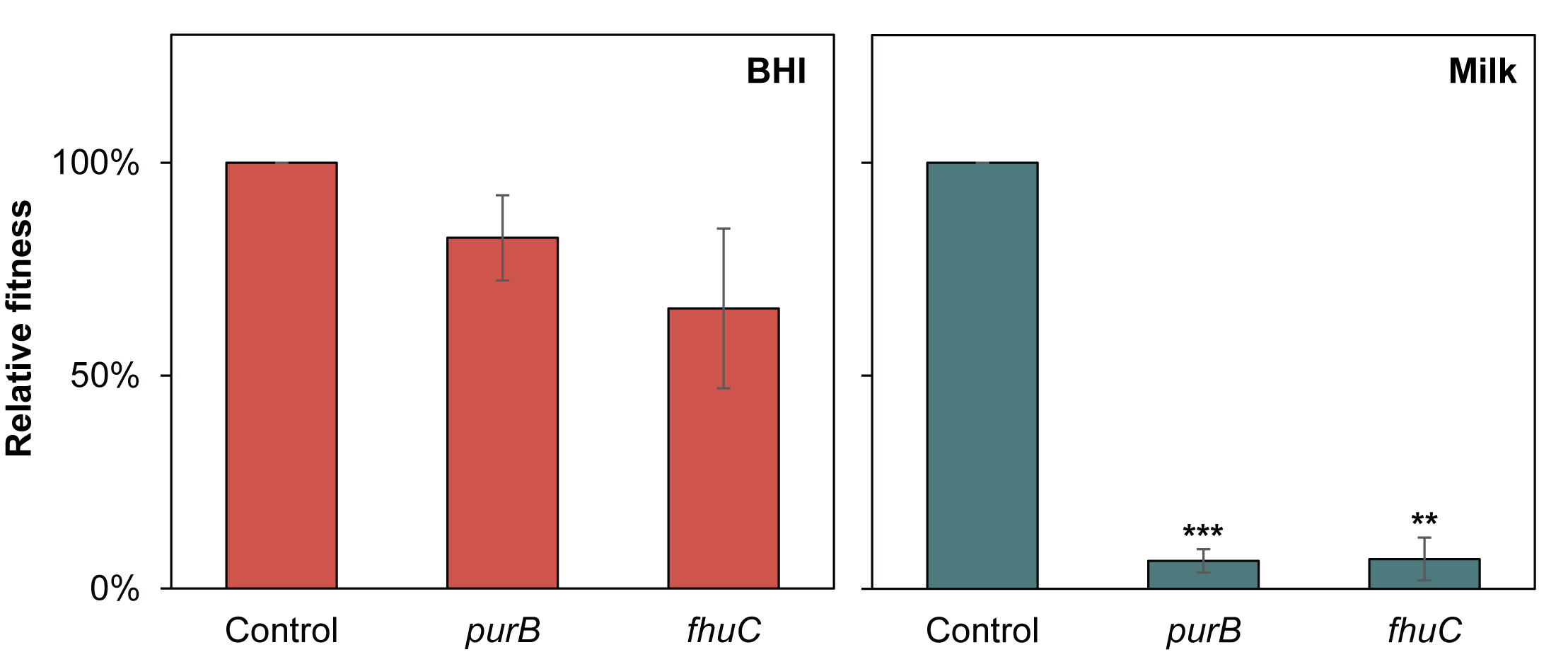

Supplement: S4 Fig — CFU/ml was calculated based on three independent spotting assays, and the relative fitness is presented as the percentage of CFU/ml of each strain relative to the control strain harboring a non-targeting sgRNA. Data represent the average and standard errors based on three independent experiments. Statistical significance was determined using two-tailed t-tests comparing each strain against the control within each condition. Significance levels are indicated as follows: * p < 0.05, ** p < 0.01, *** p < 0.001. (TIF) [file ppat.1013080.s004.tif]
