# Supplementary material for: Genome-wide analysis of fitness determinants of Staphylococcus aureus during growth in milk
Source: PLoS Pathog. 2025 Apr 9;21(4):e1013080. doi: 10.1371/journal.ppat.1013080 (PMC12011298; doi:10.1371/journal.ppat.1013080)
Supplement: S3 Table — (DOCX) [file ppat.1013080.s007.docx]

**Table S3.** List of genes less important for fitness in milk as determined by CRISPRi-seq

| **Locus tag targeted**  **(SAOUHSC)** | **Target gene(s)**^a^ | **Function/pathway** ^b^ | **Interaction**  **log_2_FC** ^c^ | **Interaction P_adj_** ^c^ |
| --- | --- | --- | --- | --- |
| _00867 | *dltXABCD* | D-alanylation of teichoic acids | 8.7 | 2,81E-08 |
| _02399 | *glmS* | Glucosamine-fructose-6-phosphate aminotransferase | 7.7 | 2,4E-06 |
| _02368 | *pyrG* | CTP synthase | 7.5 | 5,29E-06 |
| _00561 | *vraX* | Cell wall stress protein | 6.9 | 1,04E-30 |
| _00889 | *mnhABCDEFG* | Monovalent cation/H+ antiporter | 6.9 | 4,29E-05 |
| _01285 | *glnRA* | Glutamine synthetase repressor-Glutamine synthase | 6.0 | 0,000833 |
| _00781 | *hprK-lgt*-00783-00784-*trxB* | Lipoprotein | 6.0 | 2,08E-05 |
| _00953 | *ugtP-ltaA* | Glycolipid/Lipoteichic acid biosynthesis | 6.0 | 1,36E-11 |
| _01895 | *sagB* | Putative β-N-acetylglucosaminidase | 5.8 | 1,36E-19 |
| _00906 | 00906 | Fumarylacetoacetate hydrolase | 5.7 | 9,57E-05 |
| _01702 | *mtnN*-01701-01700-*aroE*-01698-*nad*-01696-*rsfS* | Chorismate biosynthesis | 5.7 | 0,002688 |
| _00934 | *spxA* | Transcriptional regulator | 5.4 | 0,000245 |
| _00788 | 00788 | GlmS regulation | 5.0 | 0,000436 |
| _00920 | *fabHF* |  | 4.7 | 0,036216 |
| _01106 | *murI*-01107-01108 | Glutamate racemace | 4.5 | 0,000521 |
| _02801 | *gtaB* | UTP-glucose-1-phosphate uridylyltransferase | 4.3 | 7,83E-08 |
| _02793 | *pgcA* | Phosphoglucosamine mutase | 4.2 | 2,39E-12 |
| _02997 | 02997-*msrA3* | Acetyltransferase -ethionine-sulfoxide reductase | 4.2 | 4,88E-18 |
| _00762 | *tagO* | Wall teichoic acid biosynthesis | 4.1 | 0,012117 |
| _01622 | 1622-*nusB-xseA*-1619-*ispA* | Endoribonuclease | 4.0 | 3,64E-07 |
| _00980 | *menA* | Menaquinone biosynthesis | 3.9 | 2,4E-06 |
| _02369 | *rpoE* | RNA polymerase, subunit | 3.8 | 0,000537 |
| _01265 | 01265 | **Metallophosphoesterase** | 3.7 | 2,19E-05 |
| _00567 | 00567 | Membrane protein | 3.6 | 0,040001 |
| _02612 | *rpiA* | Ribose-5-phosphate isomerase A | 3.4 | 1,25E-09 |
| _00189 | 0189-0190 | Membrane protein | 3.4 | 4,2E-09 |
| _A02795 | A02795 |  | 3.4 | 7,11E-06 |
| _02121 | 02121 | CamS pheromone | 3.3 | 3,77E-20 |
| _01746 | *secDF* | Preprotein translocase | 3.2 | 1,18E-08 |
| _02366 | *fbaA* | Fructose-bisphosphate aldolase | 3.1 | 0,000109 |
| _00640 | *tagA* | Wall teichoic acid biosynthesis | 3.1 | 0,004214 |
| _00474 | *rplY* |  | 3.0 | 1,91E-05 |
| _01154 | *sepF* | Cell division factor | 3.0 | 4,15E-16 |
| _01482 | *aroB* | Chorismate biosynthesis | 3.0 | 1,08E-05 |
| _01481 | *aroA* | Chorismate biosynthesis | 3.0 | 7,47E-05 |
| _00832 | *aroD* | Chorismate biosynthesis | 3.0 | 1,43E-09 |
| _01809 | *accDA* | Fatty acid biosynthesis | 2.9 | 0,000108 |
| _01495 | 01495 |  | 2.9 | 3,7E-13 |
| _01586 | *srrAB* | Two-component system | 2.9 | 8E-05 |
| _00964 | *0964-0965* | CPBP family intra-membrane metalloprotease | 2.8 | 1,42E-08 |
| _01223 | *gid* | tRNA methyltransferase | 2.6 | 0,000615 |
| _02611 | *lyrA* | CPBP family intra-membrane metalloprotease | 2.6 | 7,47E-05 |
| _01852 | *aroA2* | Chorismate biosynthesis | 2.4 | 1,39E-06 |
| _00253 | 00253 |  | 2.3 | 0,021076 |
| _01635 | *aroK* | Chorismate biosynthesis | 2.3 | 2,91E-05 |
| _00996 | 00996 |  | 2.1 | 0,010107 |
| _00787 | *rapZ-00788* | GlmS regulation | 2.1 | 7,55E-08 |
| _01022 | *thiV* | Thiamine ABC transporter | 2.0 | 1,96E-05 |
| _01405 | 01405 |  | 1.9 | 0,00587 |
| _02143 | 02143 |  | 1.9 | 0,036216 |
| _01501 | *ebpS* | Elastin binding protein | 1.8 | 8,56E-05 |
| _01821 | 01821 | DNA methylase | 1.8 | 0,016802 |
| _02899 | *02899-02900* | Aminohydrolase | 1.6 | 0,025009 |
| _01480 | 01480-01479-01478 |  | 1.6 | 0,03361 |

^a^ Genes in the same operon as the target gene is also indicated.

^b^ Functional characterization from *Aureo*Wiki [3].

^c^ L2FC (log_2_fold change) in fitness upon CRISPRi depletion and adjusted *p*-values (P_adj_) from DESeq2 analysis
