# Supplementary material for: Genome-wide analysis of fitness determinants of Staphylococcus aureus during growth in milk
Source: PLoS Pathog. 2025 Apr 9;21(4):e1013080. doi: 10.1371/journal.ppat.1013080 (PMC12011298; doi:10.1371/journal.ppat.1013080)
Supplement: S4 Table — (DOCX) [file ppat.1013080.s008.docx]

**Table S4.** Strains used in this study

| **Strain** | **Genotype and Characteristics** | **Reference** |
| --- | --- | --- |
| ***E. coli*** |  |  |
| IM08B | DH10B, Δ*dcm*, P_help_-*hsdMS*, P_N25_-*hsdS* (strain expressing the *S. aureus* CC8 specific methylation genes) | [1] |
| ***S. aureus*** |  |  |
| NCTC8325-4 | Derivative of NCTC8325, cured of prophages | [2] |
| SH1000 | *rbsU*+ derivative of strain NCTC8325-4 | [3] |
| RF122 | Bovine mastitis isolate | [4] |
| MOK023 | Bovine mastitis isolate | [5, 6] |
| MH225 | NCTC8325-4, pLOW-P_spac2_-*dcas9*, ery^r^ | [7] |
| MK1857 | MH225, pCG248-sgRNA(*pbp1*), ery^r^, cam^r^ | This work |
| MM75 | MH225, pCG248-sgRNA(*luc*). ery^r^, cam^r^ | [7] |
| MM223 | NCTC8325-4, *tetR*-Ptet-*dcas9* | This work |
| MM230 | MM223, pCG248-sgRNA(*luc*), cam^r^ | This work |
| MM267 | MM223, *tetM* | This work |
| MM268 | MM267, pCG248sgRNA(*luc*), cam^r^ | This work |
| MM269 | MM267, pCG248-sgRNA(*pbp1*), cam^r^ | This work |
| MM289 | MM267, pVL2336-sgRNA(*sarA*), cam^r^ | This work |
| MM290 | MM267, pVL2336-sgRNA(*nrdF*), cam^r^ | This work |
| MM294 | MM267, pVL2336-sgRNA(*purE*), cam^r^ | This work |
| MM295 | MM267, pVL2336-sgRNA(*purA*), cam^r^ | This work |
| MM312 | MM267, pVL2336-sgRNA(*purB*), cam^r^ | This work |
| MM313 | MM267, pVL2336-sgRNA(*thyA*), cam^r^ | This work |
| MM314 | MM267, pVL2336-sgRNA(*fhuC*), cam^r^ | This work |
| MM315 | MM267, pVL2336-sgRNA(*htsA*), cam^r^ | This work |
| MM316 | MM267, pVL2336-sgRNA(*mntA*), cam^r^ | This work |
| MM318 | MM267, pVL2336-sgRNA(*sucC*), cam^r^ | This work |
| MM342 | MM267, pVL2336-sgRNA(*clpP*), cam^r^ | This work |
| MM403 | MM267, pVL2336-sgRNA(*noc*), cam^r^ | This work |
| MM404 | MM267, pCG248-sgRNA(*SAOUHSC_01782*), cam^r^ | This work |
| MM421 | MM267, pVL2336-sgRNA(polA), cam^r^ | This work |
| MM422 | MM267, pVL2336-sgRNA(*nupC*), cam^r^ | This work |
| MM423 | MM267, pVL2336-sgRNA(*nupG*), cam^r^ | This work |
| MM424 | MM267, pVL2336-sgRNA(*murB*), cam^r^ | This work |
| MM425 | MM267, pVL2336-sgRNA(*ung*), cam^r^ | This work |
| MM426 | MM267, pVL2336-sgRNA(*SAOUHSC_02121*), cam^r^ | This work |
| MM10 | SH1000, pLOW-*dcas9*, pVL2336-sgRNA(*luc*), ery^r^, cam^r^ | This work |
| MM553 | SH1000, pLOW-*dcas9*, pVL2336-sgRNA(*purB*), ery^r^, cam^r^ | This work |
| MM554 | SH1000, p pLOW-*dcas9*, VL2336-sgRNA(*fhuC*), ery^r^, cam^r^ | This work |

cam^r^: chloramphenicol resistance; ery^r^: erythromycin resistance

**References**

1. Monk IR, Tree JJ, Howden BP, Stinear TP, Foster TJ. Complete bypass of restriction systems for major *Staphylococcus aureus* lineages. mBio. 2015;6(3):e00308-15. Epub 20150526. doi: <https://doi.org/10.1128/mBio.00308-15>. PMID: 26015493

2. Novick R. Properties of a cryptic high-frequency transducing phage in *Staphylococcus aureus*. Virology. 1967;33(1):155-66. doi: <https://doi.org/10.1016/0042-6822(67)90105-5>. PMID: 4227577

3. Horsburgh MJ, Aish JL, White IJ, Shaw L, Lithgow JK, Foster SJ. σ^B^ modulates virulence determinant expression and stress resistance: characterization of a functional *rsbU* strain derived from *Staphylococcus aureus* 8325-4. J Bacteriol. 2002;184(19):5457-67. doi: 10.1128/jb.184.19.5457-5467.2002.

4. Herron-Olson L, Fitzgerald JR, Musser JM, Kapur V. Molecular correlates of host specialization in *Staphylococcus aureus*. PLoS One. 2007;2(10):e1120. Epub 20071031. doi: 10.1371/journal.pone.0001120. PMID: 17971880

5. Keane OM, Budd KE, Flynn J, McCoy F. Pathogen profile of clinical mastitis in Irish milk-recording herds reveals a complex aetiology. Vet Rec. 2013;173(1):17. Epub 20130521. doi: 10.1136/vr.101308. PMID: 23694921

6. Murphy MP, Niedziela DA, Leonard FC, Keane OM. The in vitro host cell immune response to bovine-adapted *Staphylococcus aureus* varies according to bacterial lineage. Sci Rep. 2019;9(1):6134. Epub 20190416. doi: 10.1038/s41598-019-42424-2. PMID: 30992458

7. Liu X, de Bakker V, Heggenhougen MV, Mårli MT, Frøynes AH, Salehian Z, et al. Genome-wide CRISPRi screens for high-throughput fitness quantification and identification of determinants for dalbavancin susceptibility in *Staphylococcus aureus*. mSystems. 2024:e0128923. Epub 20240605. doi: <https://doi.org/10.1128/msystems.01289-23>. PMID: 38837392
