# Supplementary material for: Genome-wide analysis of fitness determinants of Staphylococcus aureus during growth in milk
Source: PLoS Pathog. 2025 Apr 9;21(4):e1013080. doi: 10.1371/journal.ppat.1013080 (PMC12011298; doi:10.1371/journal.ppat.1013080)
Supplement: S5 Table — (DOCX) [file ppat.1013080.s009.docx]

**Table S6.** Oligos used in this study

| **Oligo name** | **Sequence (5’ – 3’)** |
| --- | --- |
| **Construction of pMAD-*tetR*-Ptet-*dcas9*** | |
| mm40_GG_ori_up_F | GTGTCTGGTCTCCCTATCCAAGCAGTTAACGTACAAAC |
| mm32_GG_ori_up_R | GTCCAAGGTCTCGCATCGAACCCCGATGTTGTC |
| mm33_GG_Ptet_dCas9_tetR_F | GTCCAAGGTCTCCGATGCTTTTAAGACCCACTTTCAC |
| mm34_GG_Ptet_dCas9_tetR_R | GTCCAAGGTCTCCGTCATAAACGCAGAAAGGCCCAC |
| mm35_GG_ori_down_F | GTCCAAGGTCTCCTGACTCCCTTAAGACAGACCTG |
| mm36_GG_ori_down_R | GTCCAAGGTCTCCCTGCCATTGGTGGTATCGCTGTTG |
| **Construction of individual sgRNAs** | |
| *purA*_F | TATACTCCGCCAAATTGAATGGTA |
| *purA*_R | AAACTACCATTCAATTTGGCGGAG |
| *purB*_F | TATATAGTAAAGGCTACAACATCA |
| *purB*_R | AAACTGATGTTGTAGCCTTTACTA |
| *purE*_F | TATAGATACTACTTGTTTTTCGTA |
| *purE*_R | AAACTACGAAAAACAAGTAGTATC |
| *thyA*_F | TATAACTTTCTTTGTCGTTAATAG |
| *thyA*_R | AAACCTATTAACGACAAAGAAAGT |
| *sarA*_F | TATATTAACTGCTTTAACAACTTG |
| *sarA*_R | AAACCAAGTTGTTAAAGCAGTTAA |
| *htsA*_F | TATAACAAGCTGCAACTAAAAGTA |
| *htsA*_R | AAACTACTTTTAGTTGCAGCTTGT |
| *fhuC*_F | TATAATTGACGTCACTTTGCCATC |
| *fhuC*_R | AAACGATGGCAAAGTGACGTCAAT |
| *hemQ*_F | TATAATACCAACCATCTAATGTTT |
| *hemQ*_R | AAACAAACATTAGATGGTTGGTAT |
| *mntA*_F | TATAGCCGCGTACTGGTATCGATA |
| *mntA*_R | AAACTATCGATACCAGTACGCGGC |
| *nupC*_F | TATAAAGAAGAATGGTGGTTGCTT |
| *nupC*_R | AAACAAGCAACCACCATTCTTCTT |
| *nupG*_F | TATATAAAGAACCATGCTAAAAAC |
| *nupG*_R | AAACGTTTTTAGCATGGTTCTTTA |
| *polA*_F | TATATGCAAAACCATATACTGCAT |
| *polA*_R | AAACATGCAGTATATGGTTTTGCA |
| *noc*_F | TATAAACGATACGTTCAATTTGAA |
| *noc*_R | AAACTTCAAATTGAACGTATCGTT |
| *ung*_F | TATAGATATATATTTTCCCTATCA |
| *ung*_R | AAACTGATAGGGAAAATATATATC |
| *murB*_F | TATAGTATAAGTGTATCGTTTTAA |
| *murB*_R | AAACTTAAAACGATACACTTATAC |
| SAOUHSC_01782_F | TATATCAAAAATATTTTGACCTTC |
| SAOUHSC_01782_R | AAACGAAGGTCAAAATATTTTTGA |
| SAOUHSC_02121_F | TATATTCCAGCCTGGTCATCCTTA |
| SAOUHSC_02121_R | AAACTAAGGATGACCAGGCTGGAA |
