# Supplementary material for: Genome-wide analysis of fitness determinants of Staphylococcus aureus during growth in milk
Source: PLoS Pathog. 2025 Apr 9;21(4):e1013080. doi: 10.1371/journal.ppat.1013080 (PMC12011298; doi:10.1371/journal.ppat.1013080)
Supplement: S6 Table — (DOCX) [file ppat.1013080.s010.docx]

**Table S5.** Plasmids used in this study

| **Plasmid** | **Description** | **Reference** |
| --- | --- | --- |
| pCG248 | *E. coli/S. aureus* shuttle vector, amp^r^, cam^r^ | [1] |
| pCG248-sgRNA(non-target) | For constitutive expression of sgRNA, non-targeting, amp^r^, cam^r^ | [1] |
| pCG248-sgRNA(SAOUHSC_01782) | For constitutive expression of sgRNA, amp^r^, cam^r^ | This work |
| pCG248-sgRNA(*pbp1*) | For constitutive expression of sgRNA, amp^r^, cam^r^ | [1] |
| pVL2336 | *E. coli/S. aureus* shuttle vector, amp^r^, cam^r^ | [2] |
| pVL2336-sgRNA(*nrdF*) | For constitutive expression of sgRNA, amp^r^, cam^r^ | [2] |
| pVL2336-sgRNA(*noc*) | For constitutive expression of sgRNA, amp^r^, cam^r^ | This work |
| pVL2336-sgRNA(*purA*) | For constitutive expression of sgRNA, amp^r^, cam^r^ | This work |
| pVL2336-sgRNA(*purE*) | For constitutive expression of sgRNA, amp^r^, cam^r^ | This work |
| pVL2336-sgRNA(*clpP*) | For constitutive expression of sgRNA, amp^r^, cam^r^ | This work |
| pVL2336-sgRNA(*sarA*) | For constitutive expression of sgRNA, amp^r^, cam^r^ | This work |
| pVL2336-sgRNA(*purB*) | For constitutive expression of sgRNA, amp^r^, cam^r^ | This work |
| pVL2336-sgRNA(*thyA*) | For constitutive expression of sgRNA, amp^r^, cam^r^ | This work |
| pVL2336-sgRNA(*fhuC*) | For constitutive expression of sgRNA, amp^r^, cam^r^ | This work |
| pVL2336-sgRNA(*htsA*) | For constitutive expression of sgRNA, amp^r^, cam^r^ | This work |
| pVL2336-sgRNA(*mntA*) | For constitutive expression of sgRNA, amp^r^, cam^r^ | This work |
| pVL2336-sgRNA(*sucC)* | For constitutive expression of sgRNA, amp^r^, cam^r^ | This work |
| pVL2336-sgRNA(*polA*) | For constitutive expression of sgRNA, amp^r^, cam^r^ | This work |
| pVL2336-sgRNA(*nupC*) | For constitutive expression of sgRNA, amp^r^, cam^r^ | This work |
| pVL2336-sgRNA(*nupG*) | For constitutive expression of sgRNA, amp^r^, cam^r^ | This work |
| pVL2336-sgRNA(*murB*) | For constitutive expression of sgRNA, amp^r^, cam^r^ | This work |
| pVL2336-sgRNA(*ung*) | For constitutive expression of sgRNA, amp^r^, cam^r^ | This work |
| pVL2336-sgRNA*(*SAOUHSC_02121) | For constitutive expression of sgRNA, amp^r^, cam^r^ | This work |
| pMAD | Vector for allelic replacement, amp^r^, ery^r^ | [3] |
| pMAD-GG | pMAD adapted for Golden Gate cloning, amp^r^, ery^r^ | [2] |
| pFD152 | *E. coli/S. aureus* shuttle vector, *tetR*-Ptet*-dcas9* | [4] |
| pCN36 | *E. coli/S. aureus* shuttle vector, *tetM* | [5] |

cam^r^: chloramphenicol resistance; ery^r^: erythromycin resistance; amp^r^: ampicillin resistance

**References**

1. Stamsås GA, Myrbråten IS, Straume D, Salehian Z, Veening J-W, Håvarstein LS, et al. CozEa and CozEb play overlapping and essential roles in controlling cell division in *Staphylococcus aureus*. Mol Microbiol. 2018;109(5):615-32. doi: <https://doi.org/10.1111/mmi.13999>.

2. Liu X, de Bakker V, Heggenhougen MV, Mårli MT, Frøynes AH, Salehian Z, et al. Genome-wide CRISPRi screens for high-throughput fitness quantification and identification of determinants for dalbavancin susceptibility in *Staphylococcus aureus*. mSystems. 2024:e0128923. Epub 20240605. doi: <https://doi.org/10.1128/msystems.01289-23>. PMID: 38837392

3. Arnaud M, Chastanet A, Débarbouillé M. New vector for efficient allelic replacement in naturally nontransformable, low-GC-content, gram-positive bacteria. Appl Environ Microbiol. 2004;70(11):6887-91. doi: <https://doi.org/10.1128/aem.70.11.6887-6891.2004>. PMID: 15528558

4. Depardieu F, Bikard D. Gene silencing with CRISPRi in bacteria and optimization of dCas9 expression levels. Methods. 2020;172:61-75. Epub 20190801. doi: <https://doi.org/10.1016/j.ymeth.2019.07.024>. PMID: 31377338

5. Charpentier E, Anton AI, Barry P, Alfonso B, Fang Y, Novick RP. Novel cassette-based shuttle vector system for gram-positive bacteria. Appl Environ Microbiol. 2004;70(10):6076-85. doi: <https://doi.org/10.1128/aem.70.10.6076-6085.2004>. PMID: 15466553
