# Supplementary material for: Genome-wide analysis of fitness determinants of Staphylococcus aureus during growth in milk
Source: PLoS Pathog. 2025 Apr 9;21(4):e1013080. doi: 10.1371/journal.ppat.1013080 (PMC12011298; doi:10.1371/journal.ppat.1013080)
Supplement: Attachment — Submitted filename: Point-by-point_response_final.pdf [file ppat.1013080.s012.pdf]

Point-by-point response PPATHOGENS-D-24-02369

(line numbers refer to the marked-up manuscript file, where changes are shown with the Track Changes function in Word).

Reviewer #1:

The manuscript investigates the genetic fitness determinants of Staphylococcus aureus during growth in milk, employing a CRISPRi-seq system with the novel addition of aTc inducibility to enable its use in milk-based growth conditions. The study identifies 79 genes critical for growth in milk compared to BHI and shows pathways such as the purine cycle, folate biosynthesis, and metal acquisition to be of relevance. The authors validate the CRISPRi-seq dataset using individual knockdown strains and demonstrate an increased susceptibility of S. aureus to TMP-SMX during milk growth, emphasizing the importance of folate biosynthesis in this nutrient environment.

While this work is compelling and aligns well with the journal's scope, some significant shortcomings should be addressed before publication.

Response: We thank the reviewer for the feedback and suggestions to improve our manuscript.

<u>Major points:</u>

1. Relevance of Purine Cycle and Folate Biosynthesis in Milk:

The authors highlight the importance of the purine cycle and folate biosynthesis during growth in milk, suggesting these nutrients are limiting in this environment. This is unexpected, as milk is nutritionally very rich. The authors might quantify purine and folate levels in milk, including both UHT milk (as used in the study) and fresh milk. Such data would clarify whether these nutrients are indeed limited.

Response: We agree that it would be highly beneficial to quantify levels of purine and folate in milk and BHI, but we were unfortunately not able to perform this analysis. However, we have performed supplementation experiments with adenine to support this result (see below).

To further strengthen this point, supplementation of milk with purines or folate should be tested to determine whether this rescues the observed phenotypes.

Response: We have now performed experiments where we supplement adenine to milk. Supporting our findings on the importance of purine biosynthesis, these experiments showed that supplementation of adenine rescued the growth of the purB mutant in milk. See new Fig. 3C, and corresponding updates of the text in the Results section (L205 - L208) and methods section (L642-L643).

2. Use of NCTC8325-4:

- The manuscript utilizes the human isolate NCTC8325-4 but does not provide sufficient justification for its selection, particularly in the context of milk growth. This strain is known to carry genetic mutations that affect transcriptional regulation, including SigB deficiency, as

noted in https://pmc.ncbi.nlm.nih.gov/articles/PMC3786944/. SigB-deficient strains are associated with chronic udder infections, as described in https://www.nature.com/articles/s41598-019-49981-6 and https://www.frontiersin.org/journals/microbiology/articles/10.3389/fmicb.2019.02493/full. If the authors intentionally used NCTC8325-4 to model mastitis isolates, this should be explicitly discussed.

Response: We thank the reviewer for highlighting this very relevant point. We have attempted to construct a library in a mastitis-derived strains, however, our attempts so fair has not been successful due to too low transformation efficiencies in these strains (which is a pre-requisite for library construction). Therefore, we chose to use the NCTC8325-4, which was one of the strains were the CRISPRi-library was available – and which is a much-used strain in S. aureus research. Construction of libraries in clinicals is a main objective in our ongoing/follow-up work. As mentioned by the reviewer, we believe that the results obtained with NCTC835-4 are highly relevant. We have expanded the discussion of the strain in the discussion section and included the very interesting references to sigB-deficient strains found in udder-infections (L499- L510).

- The authors should address whether SigB deficiency is relevant to the phenotypes observed. The SH1000 strain, which represents a SigB-repaired variant of NCTC8325-4, could serve as a control. I would expect that SigB deficient strains show improved fitness in milk than WT strains. The fact that SigB-knowckdowns strains were not detected in the CRISPRi-seq analysis might further suggest that the used WT strain was already SigB deficient.

Response: This is a good point. We have now made knockdown strains of *purB* and *fhuC* in S. aureus SH1000 and tested the phenotypes. The effects on *purB* and *fhuC* knockdown in BHI vs milk were the same in SH1000 as in NCTC8325-4, suggesting that SigB was not critical for these phenotypes. See new Fig. S4. Nevertheless, as noted above, we have elaborated our discussion on the relevance of the NCTC8325-4 strain (L499 – L510) in the context of the sigB genotype.

- Validation of the key findings using additional bovine and human S. aureus isolates is strongly recommended to ensure the results are generalizable.

Response: Again, we fully agree on this point and have now performed assays with antibiotics (TMP-SMX and tunicamycin) on two mastitis associated strains. Indeed, the observed effects in these strains in milk vs BHI were the same as observed for NCTC8325-4. See new Fig. S3.

Note that we have also attempted to create mutants in these backgrounds to assess further phenotypes, however, we were unfortunately not been successful in these attempts.

3. Growth Defects of pur Kockdowns:

- Figure 3AB does not convincingly demonstrate growth defects for purA and purE. The differences between these strains and the wild type appear minor, and only purB shows a clear reduction in growth. Additionally, the purB colonies exhibit heterogeneity, raising questions about the method used to assess fitness.

Response: This is a good point; while both purA and purE were were clear hits in the CRISPRi-seq screen, the growth defects of the individual knockdown strains were rather minor and have rephrased accordingly;

Line 204: "… while *purA* and *purE* knockdowns exhibited only very mild growth defects"

The most likely explanation, which also applies to other genes, is that relatively mild growth defects are accentuated the CRISPRi-seq experiment due to the competitive nature of the pooled library compared to growth of single strains (as in Fig. 3AB).

- If colony-forming units (CFU) were counted from the dilution spot assay to generate the data for Figure 3B, this technique may lack precision, especially when differences between strains are relatively small (e.g., 2-fold rather than 10- or 100-fold). This likely explains the large standard deviations observed in Figure 2B. The authors should consider presenting raw CFU counts for different conditions to better assess variability and reliability of the experiment. The use of "relative fitness" in Fig. 3B is confusing, as this term is typically applied to competition assays involving co-culture of strains. For clarity, bar graphs should group strains based on growth conditions (e.g., all strains grown in milk paired together, and all strains grown in RPMI paired). This approach should be applied to Figures 4 and 5 as well.

Response: We have tested different ways to present the data, as suggested by the reviewer. We agree that grouping the data based on growth conditions (Fig. 3B, 4D, 5B) is a better way to present the data (rather than grouping by genes, as it was before), and this has now been modified. Furthermore, for Fig. 4A and 6A, we have kept the grouping by concenctration, but now plotted the data as line/scatterplot rather than bars. We would like to keep the representation with fitness relative to the control, but the "Relative fitness" has now been more clearly defined in all the figure legends. We also agree with the reviewer that the dilution spotting method may cause larger standard deviations compared to other methods, but since we've done it like this throughout the work, we would like to keep it.

Minor points:

1. Supplementary Data Files:
   - Supporting Tables S1 and S2 are not provided in the supplementary materials.

Response: We have uploaded Tables S1 and S2 as a supplementary files (.xls format).

2. Text Corrections:

- Line 42: "remain".

Response: Corrected.

- Line 64: "Colonization in staphylococci" – the meaning of this phrase is unclear and needs rephrasing.

Response: Thank you for noting this. The sentence has now been rephrased to clarify: "Additionally, growth in milk has been shown to promote the expression of biofilm-associated

proteins facilitating surface adherence and colonization ...”

- Line 72: "acid".

Response: Corrected.

- Line 169: The reference to "Table 1" seems out of place, as the analysis forming the basis of this table has not yet been described.

Response: Agree. The reference to Table 1 has been removed.

3. Experimental Replicates:

- Several figures including Figure 1C, 3B 4A and 6, indicate that only two biological replicates were included. Three biological replicates are standard for publication and should be provided.

Response: We have now repeated the experiments and the figures 3B, 4A and 6 are updated with three biological replicates. Regarding Figure 1C, this experiment had already been repeated three times. An extra sentence has been added to the Methods-section

Line 648: “All growth and antibiotic susceptibility assays were repeated at least three times.”

Reviewer #2:
I find this to be an exceptionally clearly and well written paper. The authors use the relatively new technique of CRISPRi-seq to identify Staphylococcus aureus fitness genes involved in growth in milk. They found that purine biosynthesis and other genes were essential in the milk environment compared to growth in Brain Heart Infusion medium, whereas genes involved in teichoic acid biosynthesis were less important in the milk environment. S. aureus grown in milk was shown to be more susceptible to folate biosynthesis inhibitors - trimethoprim-sulfamethoxazole. Overall, the findings may inform antibiotic therapy of mastitis.

Response: We thank the reviewer for the positive feedback on our manuscript.

Major points:

Minor points:
1. Include more description and explanation of the CRIPR technique in the Introduction to make it easier for the reader to understand.
Response: The introduction on CRISPRi and CRISPRi-seq has been updated (L87 – 94).

2. Expand on the osmoprotective effect of milk possibly being responsible for the diminished importance of teichoic acid biosynthesis in the Discussion.
Response: Our intention here was to discuss the protein that S. aureus can be protected from lysis as they are associate with milk fat globules. This has now been specified (L491-492).

3. Go down the rabbit hole of trying to compare the chemical compositions of milk and BHI (no fun I know).

Response: We agree that this would be very interesting, and we tried to interpret the data in the light of these aspects prior to submitting the paper. However, we chose not to pursue this aspect, as the exact composition of milk and BHI is not known, making it difficult to obtain reliable concentrations.

Reviewer#3:
In this study, Marli and colleagues use a genome-wide CRISPR interference sequencing (CRISPRi-seq) screen to identify genes required for growth of Staphylococcus aureus in bovine milk. The strengths of the study include the genome-wide approach to identify pathways essential for growth in milk in comparison to nutrient-rich conditions (BHI).
Another strength is the application of these data to test therapeutic potential of inhibitors of the pathways identified.
These experiments indicated that trimethoprim-sulfamethoxazole has enhanced activity for inhibiting S. aureus growth in milk.
The authors also identify that teichoic acids may have lesser importance for growth in milk compared to BHI.

Overall, the study seems well carried out with appropriate controls and provides new information relating to S. aureus growth in milk.

However, the major weakness of the study is the use of the laboratory strain 8325-4 which is a UV-treated phage-cured derivative of a human clinical isolate from an eye infection. It is unclear why the authors did not use S. aureus clinical isolates from the established bovine-associated lineages responsible for mastitis worldwide.

Bovine S. aureus lineages are adapted to the dairy niche and specialised for causing disease in dairy cattle. Therefore, the strain selected is not representative of the strains that would be found in that niche. This limits the potential for identifying genes/pathways required for survival/growth in milk.

It is feasible that some of the genes/pathways identified in strain 8325-4 to be required for growth in milk are also important for growth in bovine S. aureus lineages but this has not been examined.

Furthermore, the therapeutic potential of targeting pathways required for growth in milk has not been tested in clinically-relevant bovine S. aureus isolates.

Response: We thank the reviewer for the feedback and suggestions to improve our manuscript.

<u>Major points:</u>

- An appropriate bovine-adapted strain should be examined to determine if the same genes/pathways identified in the human-derived lab strain 8325-4 are required for growth in milk. eg using mutants/knockdowns of selected genes.

Response: This is a good point. Unfortunately, we were not able to generate the mutants in bovine strains tested. Note, however, that we did verify the involvement of the purine biosynthesis pathway and iron scavenging in the strain SH1000 (see new Fig. S4), and performed

antibiotic susceptibility test for bovine mastitis isolates (see point below).

- Test the therapeutic efficacy of the folate inhibitor antimicrobials in milk eg trimethoprim-sulfamethoxazole with relevant bovine clinical isolates representing major lineages.

Response: The effect on the TMP-SMX and tunicamycin has now been tested in bovine mastitis isolates RF122 and MOK023. The same effects as observed for NCTC8325-4 were found also for these strain. This is now shown in new Fig S3.

- The limitations of the study with regard to the strain selection and its potential to provide insights into growth in milk and adaptation to the dairy environment should be clearly stated in the Methods and in the Discussion.

Response: Agreed. We believe the discussion section is most suitable for this and an extra paragraph discussing the strain selection and the relevance of the strains used has now been added (L499-510).

Minor points:
The assertion of the authors that the study 'contributes to the understanding of bacterial adaptation in the dairy environment' is not supported as the appropriate ecologically adapted strains are not considered.

Response: We have now removed this statement to avoid any confusion on the interpretation of the results.
